# Goji Who? Morphological and DNA Based Authentication of a “Superfood”

**DOI:** 10.3389/fpls.2018.01859

**Published:** 2018-12-18

**Authors:** Sascha Wetters, Thomas Horn, Peter Nick

**Affiliations:** Molecular Cell Biology, Karlsruhe Institute of Technology, Karlsruhe, Germany

**Keywords:** Goji, superfood, food diagnostics, *Lycium barbarum*, *Lycium chinense*, molecular authentication, ARMS

## Abstract

“Goji” (*Lycium barbarum* and *Lycium chinense*) is a generic name for medical plants with a long historical background in the traditional Chinese medicine. With the emerging trend of “Superfoods” several years ago, Goji berries soon became an established product in European countries and not only are the most popular product of traditional Chinese medicine outside of China but to this day one of the symbols of the entire “Superfood” trend. However, since Goji is an umbrella term for different plant species that are closely related, mislabeling and adulterations (unconsciously or purposely) are possible. We carefully verified the identity of Goji reference plant material based on morphological traits, mainly floral structures of several inflorescences of each individual, in order to create a robust background for the downstream applications that were used on those reference plants and additionally on commercial Goji products. We report morphological and molecular based strategies for the differentiation of *Lycium barbarum* and *Lycium chinense*. The two different Goji species vary significantly in seed size, with an almost double average seed area in *Lycium chinense* compared to *Lycium barbarum*. Differences could be traced on the molecular level as well; using the psbA-trnH barcoding marker, we detected a single nucleotide substitution that was used to develop an easy one-step differentiation tool based on ARMS (amplification refractory mutation system). Two diagnostic primers used in distinct multiplex PCRs yield a second diagnostic band in a subsequent gel electrophoresis for *Lycium barbarum* or *Lycium chinense*, respectively. Our ARMS approach is a strong but simple tool to trace either of the two different Goji species. Both the morphological and the molecular analysis showed that all of the tested commercial Goji products contained fruits of the species *Lycium barbarum* var. *barbarum*, leading to the assumption that consumer protection is satisfactory.

## Introduction

A rising concern for health and an aging society seem to be important driving forces for the boom of “Superfoods” in Europe with a rapid sequence of new products entering a dynamic and further growing market. Germany ranks second (behind the United States) with respect to the import of products that are labeled as “Superfood” (Mintel, 2018). Every season new products are trending and advertised as “Superfoods.” Current trends are, for instance, basil seeds, or even varieties of cabbage, *Brassica oleracea* L., that have been in use in Germany for more than 500 years (Baumann et al., 2001) but currently experience a revival as “Superfood,” e.g., kale (*Brassica oleracea* var. *acephala*) Šamec et al., 2018. Some of these “Superfoods,” such as Chia seeds or Goji berries have in the meantime turned into established products that can be obtained in almost every supermarket or even in discounters as well. This hype of “Superfoods” is expected to continue, which can be seen in the sales numbers, that for Chia doubled from 2015 to 2016 to a volume around 23 million Euro in Germany alone (Statista, 2018a) and are still growing. Among the “Superfoods,” Wolfberries, or “Goji” rank among the leading products. “Goji” is the generic name for different plant species from the genus *Lycium*, belonging to the Solanaceae family. “Goji” represents the most popular product of traditional Chinese medicine outside of China (Chinadaily, 2018), the Western vernacular name “Goji” derives from the Chinese term *gou qi* (Potterat, 2010). In traditional Chinese medicine different plants, mainly *Lycium barbarum* L. and *Lycium chinense* Mill. are described under the umbrella term *gou qi* and have both been used for more than 2,000 years (Burke et al., 2005). Though exotic, Goji berries were already used in Europe before the introduction of the Novel Food Regulation (1997) to an extent that it did not fall under the Novel Food Legislation. However, this actually holds true only for the species *Lycium barbarum*. Several years ago, “Goji” berries were marketed as one of the first so called “Superfoods” and praised in advertisements and newspapers for miraculous properties (Latimes, 2018). Although it is already some time back that “Goji” berries became popular, they are still appreciated and bought for the perceived health effects, and continue to lead the charts for the most popular “Superfoods” (Statista, 2018b). Also in the news coverage of “Superfoods,” “Goji” is inevitably used as example and almost can be considered as symbol for the entire trend (SWR, 2018). While numerous reports refer to high levels of antioxidants, vitamins, minerals, or proteins that are claimed to account for the positive effects of such “Superfoods,” it has to be kept in mind that numerous traditional food plants harbor comparable levels of these healthy ingredients, rendering the term “Superfood” rather ambiguous. The combination of media hype with vernacular nomenclature (that had been separated from its traditional context) conceals the fact that it is often unclear, what plant is actually found in the package sold as “Goji.” Therefore, it is important to focus on the actual plants hidden behind the term “Superfood Goji.” The Flora of China distinguishes among others between *Lycium barbarum* (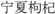, *ning xia gou qi*), *Lycium chinense* (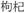, *gou qi*), and *Lycium ruthenicum* Murray (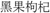, *hei guo gou qi*, also called “Black Goji”) (Efloras, 2018). These plants are thorny shrubs (common name boxthorn) with heights up to three meters, small inconspicuous purple flowers and orange to red fruits (black for *Lycium ruthenicum*) that reach up to two centimeters in length. Especially *Lycium barbarum* and *Lycium chinense* are very similar with respect to morphology and phylogenetic relationship. Moreover, they grow in the same habitat, are harvested at the same time (usually from August to October) and have both been used in traditional medicine all over East Asia since thousands of years (Potterat, 2010). Because of their important role in traditional Chinese medicine, the fruits of “Goji” have been intensively investigated with respect to their bioactive compounds (reviewed in Potterat, 2010; Amagase and Farnsworth, 2011; Masci et al., 2018), as well as for their anatomical and histochemical features (Konarska, 2018). While both species are marketed to a conspicuous extent, the Pharmacopoeia of the People’s Republic of China lists only *Lycium barbarum* as the official drug (Zhonghua Renmin Gongheguo wei sheng bu yao dian wei yuan hui, 2000), and also the Novel Food Catalogue of the EU allows the usage of only *Lycium barbarum* as food or food ingredient (Europa, 2018). Because of these legal constraints, providers of Goji Berries claim that their commercial products that are offered outside of Asia exclusively contain fruits of *Lycium barbarum* (Potterat, 2010). However, not all commercial Goji products are properly labeled with the scientific name. Usually the generic name “Goji” is accompanied by additional Western vernacular names, such as Boxthorn, Wolfberry, Chinese Wolfberry, or Matrimony Wine which does not really contribute to consumer safety (Blaschek et al., 1993). Considering how closely related these species are, this nomenclatural nonchalance and partially also ignorance will easily create mislabeling. A simple and robust one-step differentiation method based on molecular markers derived from carefully determined reference plants is needed. Such a marker has to be robust enough to be amenable to processed samples, since “Goji” is often sold as powder, and even for dry fruits it is very hard to trace back to the originating plant(s).

DNA barcoding is an important identification tool that was first used for animal identification done by amplification of the mitochondrial COI1 gene (Hebert et al., 2003). This approach has meanwhile been developed for plants as well. A number of different marker regions are commonly used, but still there is no universal region that fulfills all the desired criteria, that are: the simple amplification of the respective region with one primer pair, an adequate size to perform bidirectional sequencing without additional primer, and the condition for maximal discrimination power between species (Hollingsworth et al., 2009). The plastid psbA-trnH spacer region that is flanked by the highly conserved psbA and trnH genes is one of the most frequently used DNA barcodes and widely accepted as reliable, because of its high variance that can be used for discrimination down to the species level (Kress et al., 2005). The region has been especially valuable for phylogenetic and taxonomic studies of the genus *Solanum* from the Solanaceae family, where an estimated 96.5% (projected from a small sample range) of the species can be differentiated with this marker (Pang et al., 2012). While DNA barcoding requires sequencing, discrimination of two species with a known sequence can make use of sequence polymorphisms that can be translated into a specific pattern. Amplification of the rbcL barcode followed by restriction with appropriate enzymes has been used successfully to discriminate two Australian Myrtaceae that are commercialized under the same vernacular name “Lemon Myrtle” (Horn et al., 2012). Alternatively, such barcodes can be used to design a duplex PCR, where a diagnostic third primer located in the center of the amplicon will produce a diagnostic band in addition to the full-length barcode (Horn et al., 2014). To improve the discriminative power of this approach, the diagnostic primer is designed using the amplified refractory mutation system (ARMS) strategy, where even single base-pair substitutions in sequences can be detected by design of a 3’-end destabilized primer, which is added to the full-length primer pair. As a result, this duplex PCR yields a second, smaller amplicon that can be detected as a second band in the subsequent gel electrophoresis. Originally developed for the detection of mutations (Old, 1992), this method can be used to discriminate two closely related species using a single PCR leading to a specific fingerprint that can be visualized by gel electrophoresis. In the past, this ARMS approach had been successfully used to discriminate closely related Lamiaceae species to safeguard against adulteration in commercial products (Horn et al., 2014), or to discover adulteration of Bamboo Tea by Chinese Carnations in consequence of a false translation of a Chinese vernacular name into English (Horn and Häser, 2016). As contribution to consumer safety and quality control, we are reporting a method to validate the identity of “Goji” in commercial products. Our approach is based on carefully authenticated reference plants for different *Lycium* species that are validated by taxonomical characterization which forms the base for downstream morphological and molecular analysis of those *Lycium barbarum* and *Lycium chinense* accessions, development of a diagnostic assay, and application of this assay to clarify the identity of commercial products sold as “Superfood Goji.”

## Materials and Methods

### Plant Specimens

A set of 15 accessions of *Lycium* was analyzed in this study (Table 1). The set included two specimens of *Lycium barbarum*, one of Asian origin and one commercially available in Germany, and seven specimens of *Lycium chinense* that were all cultivated and are maintained in the Botanic Garden of the Karlsruhe Institute of Technology. Fruits from *Lycium ruthenicum* were obtained from China. To get insight into the phylogenetic relations of *Lycium barbarum* and *Lycium chinense*, four additional *Lycium* species were cultivated, including specimens of the Mediterranean (*Lycium europaeum* L.) and South American (*Lycium chilense* Bertero and *Lycium ameghinoi* Speg.) regions. In addition, the DNA of one South African specimen (*Lycium oxycarpum* Dunal) was included into the study. This set of 15 *Lycium* specimens was complemented by a set of 17 “Goji” commercial products that were obtained as dried fruits from different sources (Table 2), making in total 32 accessions.

**Table 1:** *Lycium* reference specimens, overview of identity and origin.

Accession ID	ID KIT	Taxon	Provider/Origin	GenBank ID for psbA-trnH *igs*
Lb01	1470	*L. barbarum*	Phytocomm/Taiwan	KY570934
Lb02	5548	*L. barbarum*	Phytocomm/Germany	KY570933
Lc01	5549	*L. chinense*^∗^	Fa. Ruehlemanns/Germany	KY570941
Lc02	5550	*L. chinense*^∗^	BGU Hohenheim/Germany	KY570940
Lc03	5551	*L. chinense*	IPK Gatersleben/North Korea	KY570939
Lc04	5552	*L. chinense*	IPK Gatersleben/China	KY570942
Lc05	5553	*L. chinense*	FBGU Goettingen/Germany	KY570936
Lc06	6815	*L. chinense*	NIBIO/Japan	KY570938
Lc07	6967	*L. chinense*	China	KY570937
Lr01	9176	*L. ruthenicum*	Dr. Peijie Gong/China	MH713864
Le01	7064	*L. europaeum*	BGU Lautaret/Morocco	KY570943
Le02	8213	*L. europaeum*	JBU Grenoble/Morocco	MH713865
Lo01	7067	*L. chilense*	BGU Lautaret/Argentina	KY570935
Lo02	8211	*L. ameghinoi*	JBU Grenoble/Argentina	KY570932
Lo04	8352	*L. oxycarpum*	Dr. Max Seyfried/South Africa	KY570945


*^∗^Received as *Lycium barbarum*, determined as *Lycium chinense.**

**Table 2:** Commercial Goji products obtained from different sources.

Accession ID	KIT ID	Name	Provider	GenBank ID for psbA-trnH *igs*
Gp01	8603	Goji Product 1	HANOJU Deutschland GmbH	KY683015
Gp02	8604	Goji Product 2	Krauterhaus Sanct Bernhard KG	KY683014
Gp03	8619	Goji Product 5	Grubauer’s Gewurze & Teeversand	KY683022
Gp04	8620	Goji Product 6	Feng Juan (China)	KY683021
Gp05	8621	Goji Product 7	Feng Juan (China)	KY683020
Gp06	8622	Goji Product 8	Feng Juan (China)	KY683019
Gp07	8623	Goji Product 9	Feng Juan (China)	KY683023
Gp08	8691	Goji Product 10	Edeka	KY683026
Gp09	8692	Goji Product 11	Alnatura	KY683025
Gp10	8693	Goji Product 12	Edeka	KY683024
Gp11	8694	Goji Product 13	Phytocomm	KY683017
Gp12	8695	Goji Product 14	Phytocomm	KY683016
Gp13	8696	Goji Product 15	VitFrisch	KY683018
Gp16	9310	Goji Product 16	Dr. Peijie Gong	MH713866
Gp17	9293	Goji Product 17	Dr. Peijie Gong	MH713867
Gp18	9307	Goji Product 18	Jumbo store (Netherlands)	MH713868
Gp19	9308	Goji Product 19	Raw organic food	MH713869


### Identification of Reference Specimens

The identity of specimens was verified by taxonomical identification using appropriate taxonomic keys (Flora of China (Efloras, 2018), Schmeil/Fitschen: The Flora of Germany and the neighboring countries, 95th edition (Schmeil and Fitschen, 2011). In detail, we documented floral traits (i.e., pubescense of corolla, undulation of calyx, length of corolla tube) from 30 inflorescenses of each of the *L. barbarum* and *L. chinense* accessions to eliminate errors in determination that might occur when looking only at a single flower. The observed characteristics were documented digitally (Stereolupe 420, Leica, Bensheim, Germany).

### Seed Analysis

Fruits of the cultivated plants were harvested and the seeds phenotyped quantitatively. 30 seeds of each *Lycium barbarum, Lycium chinense* and *Lycium ruthenicum* accession, as well as from all of the commercial “Goji” products were excised from at least five different fruits and digital images recorded (Stereolupe 420, Leica, Bensheim, Germany). The digital images of the seeds were analyzed using the program SmartGrain (Tanabata et al., 2012). With this software many parameters like area size or length-to-width ratio of seeds can be measured, after the digital image of the seeds is loaded into the program, and the scale bar is calibrated by the “set scale” tool. SmartGrain automatically detects the objects of the image, or the seeds can be picked manually as well. To evaluate and illustrate the obtained data as boxplots R Studio version 3.2.0 was used.

### DNA Barcoding

DNA from fresh leaves of reference plants (using 60 mg of starting material) and dried fruits of commercial products (using 120 mg of starting material) was isolated using the Invisorb^®^ Spin Plant Mini Kit (Stratec Biomedical AG). The quality and quantity of isolated DNA was evaluated by spectrophotometry (NanoDrop, Peqlab), and DNA concentration was diluted to 50 ng/μl to be used as template in PCR.

A 30 μl reaction volume containing 20.4 μl nuclease free water (Lonza, Biozym), onefold Thermopol Buffer (New England Biolabs), 1 mg/ml bovine serum albumin, 200 μM dNTPs (New England Biolabs), 0.2 μM of forward and reverse primer (see Primer list, Table 3), 100–150 ng DNA template and three units of Taq polymerase (New England Biolabs) was used to amplify the marker sequences.

**Table 3:** Primer list.

Name	5′→ 3′ sequence	Purpose	Design
psbA*^u^*	GTTATGCATGAACG TAATGCTC	Amplification of the psbA-trnH spacer region	Sang et al., 1997
trnH*^u^*	CGCGCATGGTGGAT TCACAATCC		Tate and Simpson, 2003
LB_265T_fw*^d^*	GCATTTATTCATGAT TGAGTATTCTATTCTT	Additional diagnostic band in *Lycium barbarum*	Current study
LC_265T_fw*^d^*	CATTTATTCATGATT GAGTATTCTATTCTG	Additional diagnostic band in *Lycium chinense*	Current study


*U, universal primers; d, diagnostic primer for ARMS. For the diagnostic primers the decisive nucleotide is underlined.*

Thermal cycler conditions for the amplification of the psbA-trnH intergenic spacer region included initial denaturation at 95°C for 2 min; following 33 cycles at 94°C for 1 min, 56°C for 30 s, 68°C for 45 s; ending with an extension of 68°C for 5 min.

The PCR was subsequently evaluated by agarose gel electrophoresis using NEEO ultra-quality agarose (Carl Roth, Karlsruhe, Germany). DNA was visualized using SYBRsafe (Invitrogen, Thermo Fisher Scientific, Germany) and blue light excitation. The fragment size was determined using a 100 bp size standard (New England Biolabs). Amplified DNA was purified for sub’sequent sequencing using the MSB^®^ Spin PCRapace kit (Stratec). Sequencing was outsourced to Macrogen Europe (Netherlands) or GATC (Germany).

The quality of the obtained sequences was examined with the program FinchTV Version 1.4.0^1^. To get a more robust result, the marker region was sequenced from two directions. The resulting two sequences were merged for each accession.

### Phylogenetic Analysis

For the sequence alignment and the phylogenetic analysis the program MEGA7 (Version 7.0.14) with the integrated tree explorer was used (Kumar et al., 2016). The Sequences were aligned using the Muscle algorithm of MEGA7 (Edgar, 2004). Alignments were trimmed to the first nucleotide downstream of the forward primer and the nucleotide preceding the reverse primer. With the same software, the evolutionary relationships were inferred by using the neighbor-joining algorithm with a bootstrap value that was based on 1,000 replicates (Felsenstein, 1985; Saitou and Nei, 1987). The species *Nolana werdermannii* was chosen as an outgroup, since the genus *Nolana* is a sister taxon to the Lycieae (Levin and Miller, 2005). The psbA-trnH spacer region sequence for *N. werdermannii* (GenBank: FJ189604) was obtained from the NCBI database.

### ARMS Diagnostics

A single nucleotide difference in psbA-trnH intergenic spacer sequences of *L. barbarum* and *L. chinense* was used to design a diagnostic primer to clearly discriminate these two closely related species in an one-step duplex-PCR protocol.

The primer was designed with the Primer3Plus webtool (Untergasser et al., 2012). A thymine in position 265 of the psbA-trnH multiple sequence alignment in *Lycium barbarum* is substituted by a guanine in the other *Lycium* species. Thymine was placed at the 3′-end of the diagnostic primer and an additional nucleotide was exchanged in the 3′-region, to prevent the binding of the primer to the other *Lycium* species. With this design, this diagnostic primer (LB_265T_fw) should only be able to bind to the *Lycium barbarum* accessions. Since the region surrounding this nucleotide substitution is very AT-rich, the primer length had to be increased to 31 nucleotides in order to reach an appropriate annealing temperature.

Based on the same strategy, a second diagnostic primer (LC_265T_fw) was designed that should only bind to the psbA-trnH spacer region template of *Lycium chinense*.

These diagnostic primers were then used in combination with the universal psbA and trnH primers (see primer list, Table 3) in distinct duplex-PCRs. Usage of either LB_265T_fw or LC_265T_fw ought to yield an additional second diagnostic band of 290 bp in only one of the species while in the other species only the full-length amplicon with a length of 546 base pairs would be visible after the gel electrophoresis. We tested this prediction and validated the predicted readout experimentally (Figure 6).

## Results

### Morphology of Floral Organs Clearly Delineate the Two Main “Goji” Species

**FIGURE 1:**
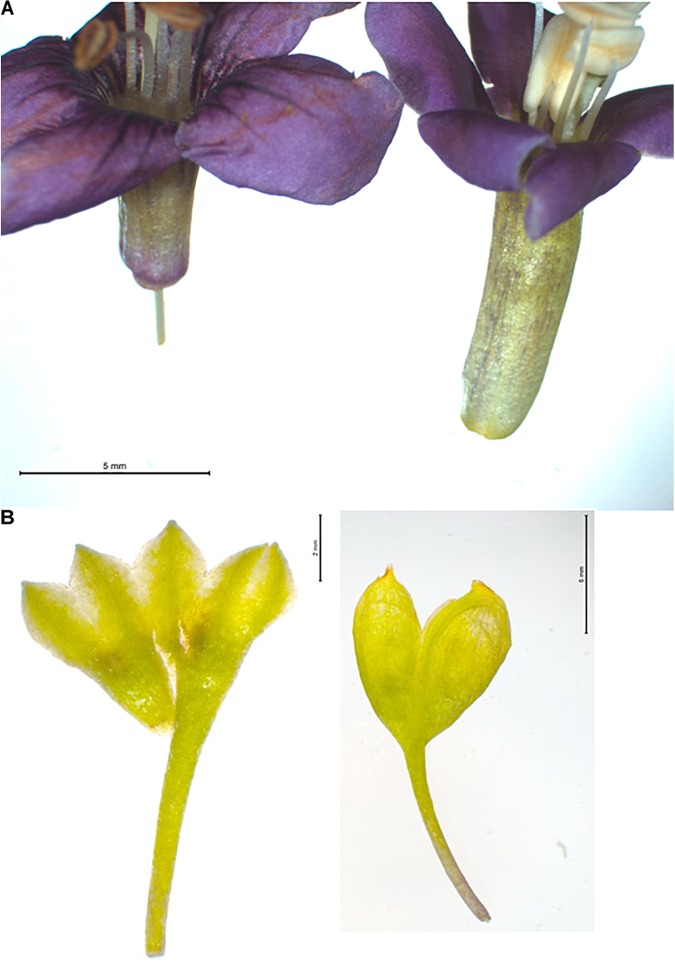
Two morphological traits to differentiate *Lycium barbarum* and *Lycium chinense* based on floral traits. **(A)** Corolla of *Lycium chinense* (left) and *Lycium barbarum* (right). The flora of china mentioned difference in corolla tube length is clearly visible; for *Lycium barbarum* the corolla tube is obviously longer than the corolla lobes. **(B)** Opened and flattened calyx of *Lycium chinense* (left) and *Lycium barbarum* (right). *Lycium chinense* is usually three to five lobed, whereas *Lycium barbarum* has only two lobes. Compared to the corolla tube length this trait is not 100% consistent.

**FIGURE 2:**
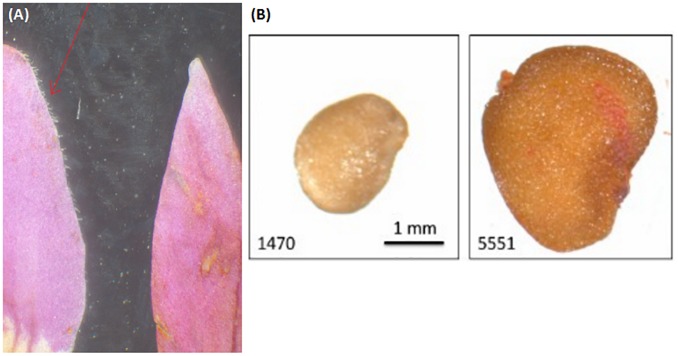
**(A)** Comparison of corolla lobe pubescence in *Lycium chinense* (left) and *Lycium barbarum* (right). The corolla lobes of the latter are glabrescent, while *Lycium chinense* corolla lobes are finely haired. **(B)** Difference in seed size can be seen with the bare eye in some cases, with significant smaller seeds of *Lycium barbarum*. See Figure 3.

To assess the validity of morphological traits that are used to delineate the main species behind “Goji,” we assessed three morphological features that are used to discriminate *Lycium barbarum* and *Lycium chinense* (Reference Flora of China) across 30 inflorescences collected from the reference plants (Figures 1, 2). An elongated corolla tube and glabrescence of corolla blades as distinctive traits of *Lycium barbarum* (Figure 1A) were consistently found over all investigated 30 inflorescenses collected from each of the two reference plants. In contrast, for all reference plants of *Lycium chinense* all corolla blades were densely pubescent at the margin (Figure 2A), and the corolla tube was distinctively shorter than the corolla lobes (Figure 1A). Thus, both of these traits could be validated to differentiate *Lycium barbarum* and *Lycium chinense* specimens. Opposed to this, the third feature reported to discriminate the two species, the number of calyx lobes (Figure 1B), was not consistent over all flowers of *Lycium chinense*. Depending on the accession, between 6.7% (in accessions 5551 and 6815) and 23.3% (in accession 5550) of the *Lycium chinense* flowers had a two-lobed calyx, which is usually characteristic for *Lycium barbarum*. In fact, without any exception, all flowers of our *Lycium barbarum* accessions had a calyx that was two-lobed. The Flora of China additionally lists different varieties of *Lycium barbarum* and *Lycium chinense*, respectively. However, all of the available reference plants were determined as either *Lycium barbarum* var. *barbarum* or *Lycium chinense* var. *chinense.*

### Seed Size Can Be Used to Discriminate the Two “Goji” Species

**FIGURE 3:**
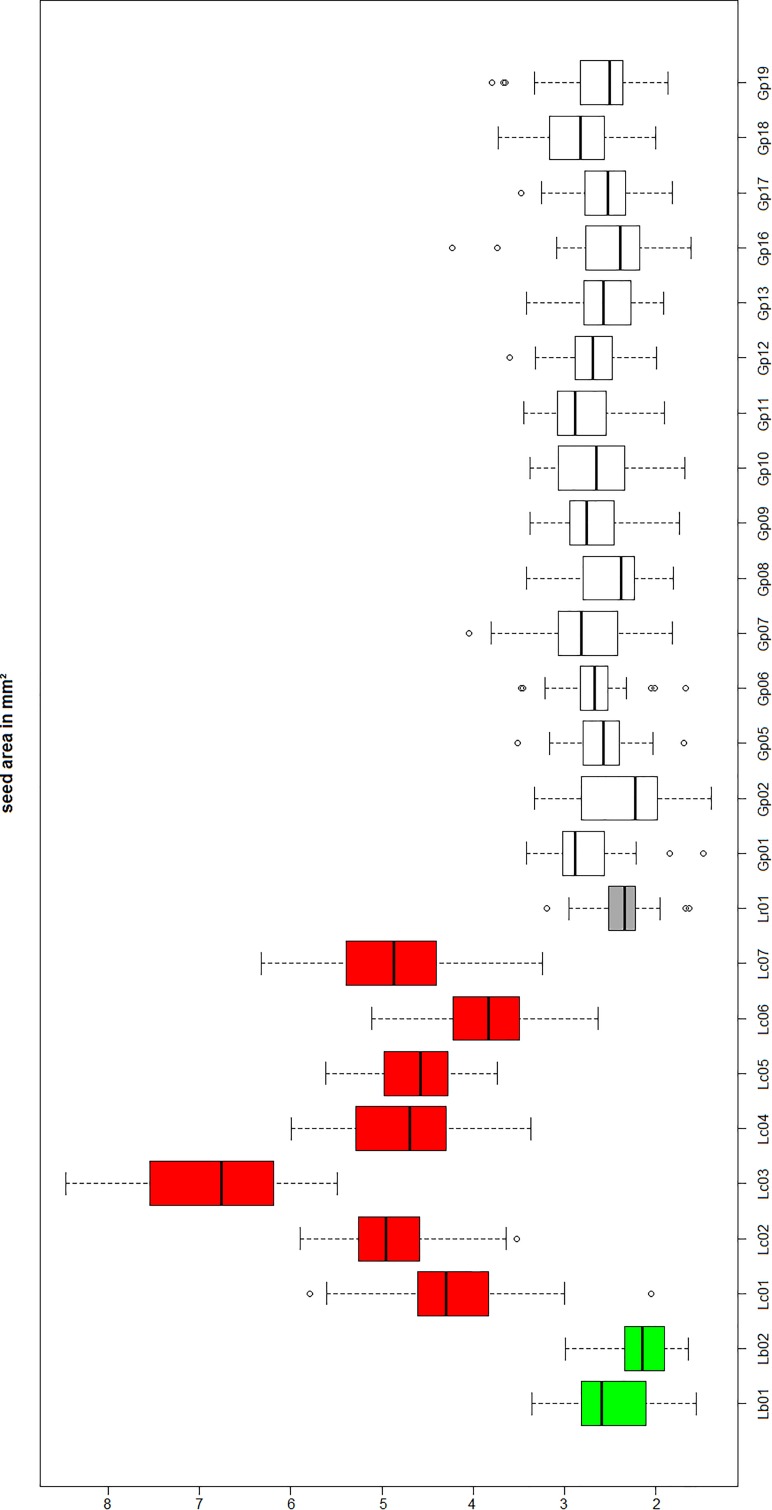
Lb, *Lycium barbarum* var. *barbarum*; Lc, *Lycium chinense* var. *chinense;* Lr, *Lycium ruthenicum;* Gp, commercial Goji product. Seed size evaluation of the reference plants *Lycium barbarum* var. *barbarum* (green), *Lycium chinense* var. *chinense* (red) and *Lycium ruthenicum* (dark gray). Those values are compared to the commercial Goji products (white). The median of the *Lycium barbarum* var. *barbarum* reference plants is between 2 and 3 mm^2^, whereas the *Lycium chinense* var. *chinense* seeds are significantly bigger in area size with a median between 4 and 6 mm^2^. *Lycium ruthenicum* seeds are smaller again, with a median slightly bigger than 2 mm^2^. The commercial Goji products have boxplots medians ranging from 2 and 3 mm^2^, similar to *Lycium barbarum* and *Lycium ruthenicum*.

Since “Goji” is traded as fruit, we were searching for morphological traits that can be inspected in fruits and allow to discriminate the two types of “Goji” berries. We noted that the seeds of *Lycium barbarum* var. *barbarum* and *Lycium chinense* var. *chinense* differ significantly in size (Figure 2B), and we quantified this trait by measuring cross-section areas (Figure 3). The two available accessions of *Lycium barbarum* var. *barbarum* showed cross sections that were less than half of those seen in the seven investigated *Lycium chinense* var. *chinense* (<2.5 mm^2^ as compared to almost 5 mm^2^). Although the intraspecific variation in *Lycium chinense* var. *chinense* was more pronounced, even the accessions with smaller seeds were clearly bigger than seeds from *Lycium barbarum* var. *barbarum* plants. The single available accession of *Lycium ruthenicum* had small seeds that are comparable to the values of the two *Lycium barbarum* var. *barbarum* sizes, but it is easy to delineate those fruits, due to their black pericarp. With an average boxplot median of 2.62 mm^2^ all the investigated commercial Goji products had seed sizes that were comparable to *Lycium barbarum* var. *barbarum*, with low variance between the different commercial samples; the lowest median was Goji product 2 (2.22 mm^2^), and the highest median was Goji product 19 (2.88 mm^2^), suggesting that these “Goji” products indeed contained *Lycium barbarum.*

### psbA-trnH Spacer Phylogeny Clearly Distinguishes *Lycium* Species

**FIGURE 4:**
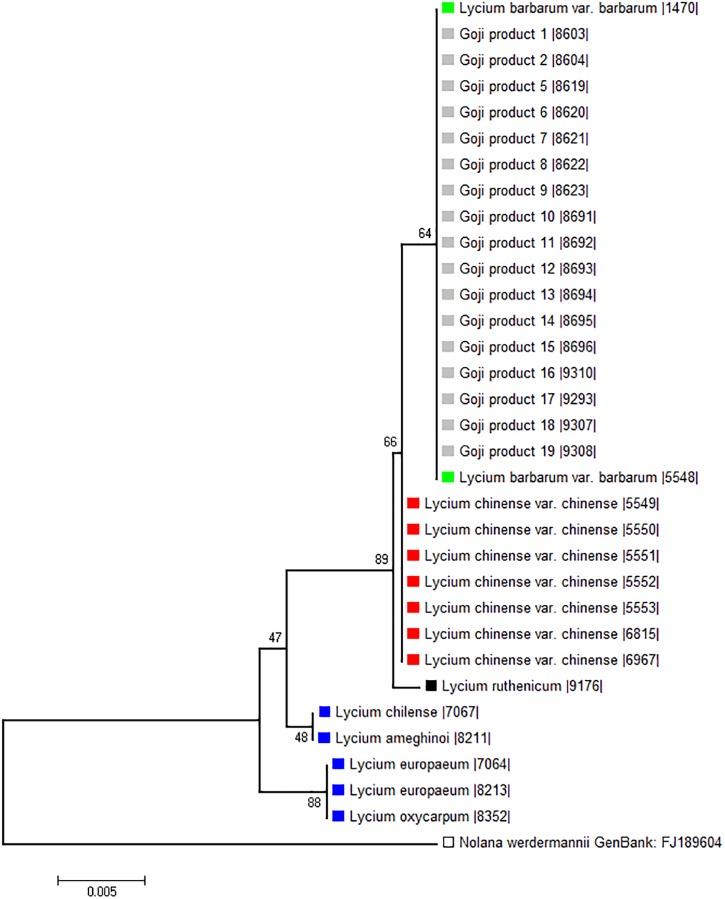
Phylogenetic tree based on psbA-trnH spacer region sequences of morphological identified *Lycium* reference plants and commercial Goji products. The evolutionary history was inferred using the Neighbor-Joining method (Saitou and Nei, 1987). The percentage of replicate trees in which the associated taxa clustered together in the bootstrap test (1,000 replicates) are shown next to the branches (Felsenstein, 1985). There were a total of 532 positions in the final dataset. *Nolana werdermannii* was chosen as outgroup. The reference plants of *Lycium barbarum* var. *barbarum* (green), *Lycium chinense* var. *chinense* (red) and *Lycium ruthenicum* (black) are labeled with colored squares. The evaluated commercial Goji products are labeled with light gray squares. To put the three different “Goji” species into a context of the *Lycium* genus, species from South America (*Lycium chilense* and *Lycium ameghinoi*, yellow) and Europe (*Lycium europaeum* and *Lycium oxycarpum*, blue) were included into the phylogenetic analysis. The *Lycium barbarum* var. *barbarum* reference plants cluster with the Goji products, while *Lycium chinense* var. *chinense* (that is closest related to *Lycium barbarum*) and *Lycium ruthenicum* have their own clusters.

**FIGURE 5:**
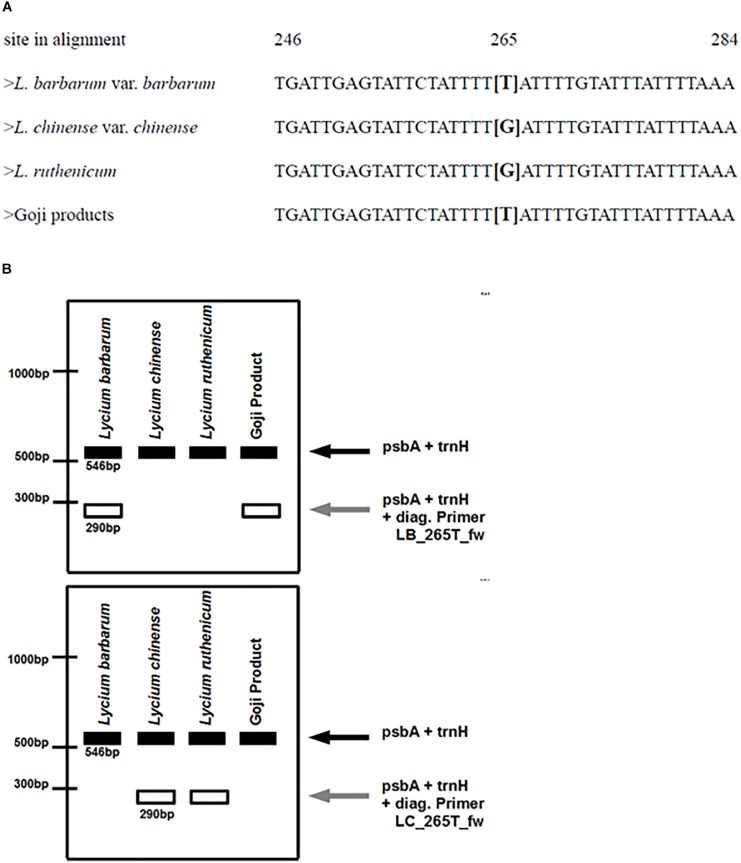
**(A)** Partial depiction of the multiple sequence alignment of different reference plants. The decisive nucleotide that is used for designing the diagnostic primers is located at site 265 in the alignment of the psbA-trnH spacer regions of the respective samples. *Lycium barbarum* var. *barbarum* and the commercial Goji products have a thymine at this site, whereas *Lycium chinense* var. *chinense* and *Lycium ruthenicum* have a guanine. **(B)** Predicted banding pattern of the psbA-trnH spacer region fragment and the diagnostic fragments after the ARMS-based multiplex PCR. Left: using the diagnostic primer for tracing *Lycium barbarum* var. *barbarum*. Right: using the diagnostic primer for tracing *Lycium chinense* var. *chinense* and *Lycium ruthenicum*.

To probe the phylogenetic relationship of the three different “Goji” species (*Lycium barbarum* var. *barbarum, Lycium chinense* var. *chinense* and *Lycium ruthenicum*) and the tested commercial products (abbreviated as Gp in Figure 4) in comparison to *Lycium* accessions from Europe and the New World, we used a 510-bp region of the psbA-trnH marker including the variable intergenic spacer (Figure 4). Although this universal psbA-trnH spacer region is one of the most variable DNA barcodes, we found only small differences between those three obviously closely related species. The only difference between *Lycium barbarum* var. *barbarum* and *Lycium chinense* var. *chinense* was one nucleotide substitution at site 265 with a thymine for *Lycium barbarum* var. *barbarum* and a guanine for *Lycium chinense* var. *chinense* and *Lycium ruthenicum* (Figure 5A). As already seen for seed size (Figure 3), the commercial “Goji” products all clustered with *Lycium barbarum* by sharing the informative thymine at position 265. The three Asian species are closely related to each other and clearly clustered separately from the two tested species from South America, as well as from the two species from Europe and South Africa (Figure 4). *Lycium ruthenicum* had an additional substitution from thymine to guanine at site 358 and an additional nine nucleotide insert at site 450, that was neither present in *Lycium barbarum*, nor in *Lycium chinense*. All accessions of *L. barbarum* var. *barbarum* and *L. chinense* var. *chinense* were identical within the respective species. The variations were exclusively interspecific. This single nucleotide substitution was used to design a strategy based on ARMS to discriminate *Lycium barbarum* from *Lycium chinense*.

### A One-Step Protocol Based on ARMS Allows to Differentiate the Two Main “Goji” Species

To develop an assay that allows an easy one-step discrimination of the two main “Goji” species in unprocessed commercial samples, we used the single nucleotide polymorphism at position 265 of the *psbA-trnH* spacer (Figure 5A) to apply an ARMS based-strategy. Two diagnostic primers – LB_265T_fw and LC_265T_fw – with complementary readouts were designed (Figure 5B).

**FIGURE 6:**
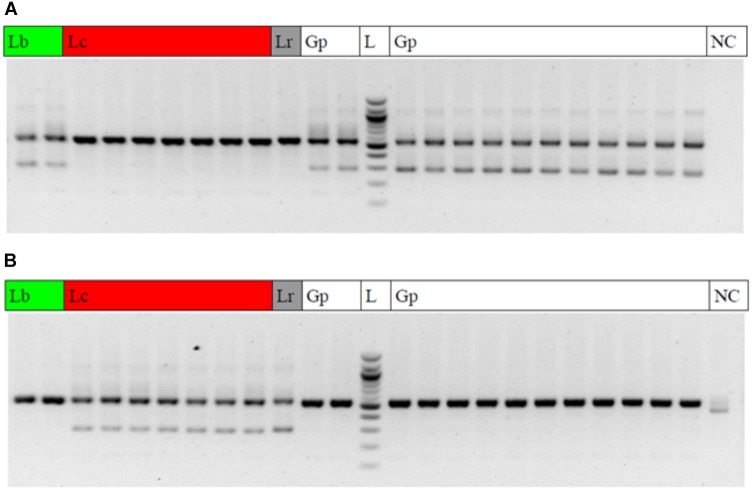
Gel electrophoresis result of the multiplex ARMS PCRs. Lb, *Lycium barbarum* var. *barbarum;* Lc, *Lycium chinense* var. *chinense*; Lr, *Lycium ruthenicum;* Gp, commercial Goji product; L, 100 bp ladder; NC, negative control. **(A)** PCR with the universal psbA and trnH primers and the additional LB_265T_fw diagnostic primer to trace *Lycium barbarum* var. *barbarum*. A clear double band occurs with the *Lycium barbarum* var. *barbarum* reference plants (lines 1 and 2) and all of the tested Goji products (Lines 11, 12, and 14–24). The second (diagnostic) band is absent in *Lycium chinense* var. *chinense* reference plants (lines 3–9) and *Lycium ruthenicum* (line 10). Line 13 is a 100 bp ladder. The last lane is the negative control. **(B)** PCR with the universal psbA and trnH primers and the additional LC_265T_fw diagnostic primer to trace *Lycium chinense* var. *chinense*. When using this additional primer a second band is visible for all the *Lycium chinense* var. *chinense* reference plants (lines 3–9) and *Lycium ruthenicum* (line 10). This band is absent in *Lycium barbarum* var. *barbarum* and the commercial Goji products.

The diagnostic LB_265T_fw primer yielded clear diagnostic bands with a fragment length of 290 base pairs at the two *Lycium barbarum* var. *barbarum* accessions and all of the Goji products (Figure 6A), while this diagnostic band was absent in *Lycium chinense* var. *chinense* and *Lycium ruthenicum*. Conversely, adding the LC_265T_fw diagnostic primer in a duplex-PCR, the diagnostic 290-band was present in all the *Lycium chinense* var. *chinense* accessions and the *Lycium ruthenicum* sample, but absent in the *Lycium barbarum* var. *barbarum* reference plants and the commercial Goji products (Figure 6B).

## Discussion

In the current work, we have developed an one-step assay to discriminate two closely related, but distinct species of *Lycium* that are used in traditional Chinese medicine and are currently booming in Europe and US as so called “Superfood” under the common vernacular name of “Goji berries.” Based on validated plant material, we have developed morphological and molecular traits that allow to differentiate between both species, and we have developed a robust one-step PCR-based assay that can reliably identify any given unprocessed commercial product as either *Lycium barbarum* or *Lycium chinense*.

### Identity Matters: Any Assay Is Only as Good as the Reference Material Is Reliable

It is a time where the possibilities of plant molecular biology appear to be overwhelming, including huge throughput of whole-genome sequencing, mapping of gene regulatory networks and phylogenomics; however, a rather ancient nevertheless essential discipline of botany starts to have its renaissance (Ledford, 2018). Although it is often considered as trivial task, to actually look at and morphologically determine the plant that is the subject of the study, this task is far from trivial. Surveys of misdeterminations and mislabeling in herbaria, botanical gardens, and germplasm collections show that between 10 and 20% of accessions are not what they are supposed to be (Goodwin et al., 2015).

Even for DNA barcoding, the correct determination of the used reference plant material is a necessary precondition for the validity of the outcome. This requirement is even more important for medicinal plants or plants that are *en vogue* because of their promised health effects. The power of the determination key is crucial to have a robust background for all subsequent experiments. Unfortunately in many keys terms with a low degree of certainty like “about” or “usually” are used, that can be interpreted differently by different taxonomists. This is also true for the otherwise high-quality Flora of China, that was mainly used in this study. For the discrimination of *Lycium barbarum* and *Lycium chinense* terms such as “usually 2-lobed” can be found in this key. To avoid uncertainties in determining a plant species, different morphological traits should be applied, if possible. The Flora of China lists three different traits to distinguish between the two *gou qi* species, *Lycium barbarum* and *Lycium chinense*.

Regarding the close relation of those two species, the common usage of both species in traditional Chinese medicine and the expectation of consumers to get the correct plants in the purchased products, the importance of correct determination is undoubtedly urgent. For the discrimination of closely related plant species, the evaluation of floral morphology traits should be central, since they are most likely linked with the reproductive isolation between these species. With the combination of the different floral traits, corolla pubescence, length of corolla tube and the number of calyx lobes, an impeccable discrimination of the two widely used Goji species could be done for the available reference plants. However, the corolla traits were more robust and therefore more reliable compared to those of the calyx, since there was variability in the features of the calyx in a considerable number of investigated inflorescences of *Lycium chinense*. The importance of evaluating an appropriate number of flowers becomes obvious, because phenomena like phenotypic plasticity or changes due to environmental factors can influence the appearance of single plant organs. For instance, differences in the velocity of polar auxin transport as they might occur depending on light quantity and quality are expected to modulate the number of apices in the primordial whorl committed to form the calyx (Reinhardt et al., 2003). However, the possibility that the morphospecies *Lycium chinense* might comprise several genetically isolated cryptospecies, should also be kept in mind.

The seed analysis revealed significant differences in the area size of *Lycium barbarum* var. *barbarum* and *Lycium chinense* var. *chinense* specimens. Thus, besides the prior described differences in the reproductive organs the two species can be distinguished by the size of their dispersal units. All the seeds of the tested commercial Goji products had similar boxplots medians compared to the two *Lycium barbarum* var. *barbarum* reference plants, wherefore we conclude that all the investigated commercial Goji products are actually fruits of the *ningxia gou qi* (*Lycium barbarum*). The boxplot median of the commercial products is comparable to *Lycium ruthenicum* as well; however, since the fruits of *Lycium barbarum* and *Lycium chinense* differ in size and especially in color from *Lycium ruthenicum*, surrogation by this species is unlikely.

### “Goji Berries” Are *Lycium barbarum*

The evaluated commercial Goji products of this study were obtained from a many different companies and different geographic origin. Nevertheless, all of the investigated products seem to originate from *Lycium barbarum* var. *barbarum* plants. Asian companies claim to sell only the *ningxia gou qi* (*Lycium barbarum*) as “Goji” in commercial products outside of China. Our results suggest that the consumer protection seems to be quite satisfactory in this respect, and that the “correct” products are sold on the German market. However, the pronounced difference in price for otherwise comparable “Goji” products indicates that consumer protection has to consider additional aspects of quality assessment. In this context, it should be noted that in China, “Goji” is marketed in different quality grades termed “super,” “king,” “special,” and “Grade A,” (EzineArticles, 2018) whereby the grade is linked with berry size. In other words, berries of *Lycium chinense* would be sold at higher prize. Thus, the fact that only the small *Lycium barbarum* berries were found in the commercial products sampled in Germany might therefore have a different explanation from a efficient system of consumer protection: the smaller berries from *Lycium barbarum* are less attractive for consumers in China (except for the few knowledgeable professionals that know about their medicinal value) and therefore are preferentially exported to Europe and the United States, where they still can be sold at high price. While the commercial Goji products as well as the *Lycium barbarum* var. *barbarum* reference plants show a fairly homogenous seed size, there is significant intraspecific variation in seed size among the accessions of *Lycium chinense* var. *chinense*. Since the measured seeds were chosen randomly and the sample size was sufficient, the likelihood that this variation is caused by sampling bias is rather low, which means that the differences in size must have genetic or developmental reasons. There are some reports that the chromosome number of *Lycium chinense* varies from 2*n* = 24 to 36 or even 48 (Tropicos, 2018), which would mean that there exist cryptospecies within *Lycium chinense*. This might also be a possible factor for the observed variability seen in calyx lobing between the different accessions of this species. The enlargement of seed size in *Lycium chinense* might therefore be linked with allopolyploidy. However, for the closely related Asian Goji species the literature regarding karyotypes is surprisingly scarce.

During the past years, the phylogenetic relations within the genus *Lycium* and its biogeographic background has been intensively studied. One of the surprising outcomes was the paraphyletic nature of *Lycium* (Fukuda, 2001; Levin and Miller, 2005). Different barcoding marker regions were used and combined for the genus to which Goji belongs, ranging from the more common rbcL, matK, trnF–trnH, psbA-trnH to conserved ortholog sequences (COS) regions (Levin and Miller, 2009). For our morphologically validated species we choose the sequences of the highly variable chloroplast psbA-trnH spacer region to find small differences in sequences that can be utilized for a simple one-step test to discriminate *Lycium barbarum* and *Lycium chinense*. However, we wanted to link our data into the context of the *Lycium* genus as well. We found three well separated clades corresponding to the three biogeographic regions. The East Asian “Goji” species were closely related, and formed a separate clade distinct from the other Old-World and the clade from New-World species of *Lycium*. Of course, for a biogeographic and in-depth phylogenetics approach many independent regions should be evaluated for a large set of species to receive a robust tree. However, as mentioned above this has been done extensively and our main goal was to find informative single-nucleotide polymorphisms that can be used to differentiate between the two “Goji” species *Lycium barbarum* and *Lycium chinense* by a one-step ARMS assay. The reliance on DNA based-methods is crucial, since the flowers of a commercial Goji product can obviously not be traced back, and some commercial Goji products are processed as powders as well.

### Authentication by ARMS Provides an Innate Positive Control for DNA Quality and PCR Quality

Based on the single nucleotide substitution between *Lycium barbarum* and *Lycium chinense* two diagnostic ARMS primers could be designed to trace either of the species. In both of the *Lycium barbarum* var. *barbarum* reference plants using the LB_265T_fw primer, and in all of the seven *Lycium chinense* var. *chinense* reference primer using the LC_265T_fw primer, the desired second diagnostic band could be amplified in distinct multiplex PCR approaches. The results of this molecular approach strengthen the data of the seed analysis, because for all commercial Goji products there was an additional diagnostic band, when using the LB_265T_fw primer. Thus, all of the investigated commercial Goji products were authenticated morphologically and by molecular markers to be *Lycium barbarum* var. *barbarum*. The advantage of this ARMS approach is the implemented positive control of the universal psbA-trnH spacer region band in the gel. This control displays that the extracted DNA is actually of good quality and, more importantly, that the region of interest is present and the discrimination occurs exclusively because of the nucleotide substitution between the two species. Apart from the practical application of the discrimination for two closely related species of commercial importance, we’d like to highlight the importance of morphology and taxonomy and the endangered art of looking at plants in order to really understand them.

While considerable effort has been invested into the evaluation of bioactive compounds of Goji (Chang and So, 2008), to our knowledge this is the first case, where a robust one-step discrimination for these closely (from an evolutionary, cultural-historic and medicinal point of view) related “Goji” species has been developed. Previous studies have used RAPD fingerprinting and the downstream Sequence characterized amplified region (SCAR) strategy (Zhang et al., 2001; Sze et al., 2008). A disadvantage of RAPD in authentication is its limited reproducibility and reliability as fingerprint patterns are strongly dependent on sample quality and DNA integrity. Despite these drawbacks RAPD is a rapid and cost effective method that continues to be used today (Krishnan et al., 2017), especially for authentication approaches combined with downstream SCAR markers (Mei et al., 2017). RAPD-based SCAR markers yield reproducible binary results, and this method has been used successfully for “Goji” (Sze et al., 2008), ginseng (*Panax ginseng* C.A. Mey.) (Wang et al., 2001), saffron (*Crocus sativus* L.) (Torelli et al., 2014), and others. The validation by SCAR at first sight seems easier to interpret, because only a single band has to be assessed. However, missing bands after gel electrophoresis could also be caused by problems with DNA purity or integrity, or likewise with suboptimal conditions of the PCR. Both problems are avoided by the ARMS approach, because the full-length amplicon serves as an inbuilt positive control to calibrate presence or absence of the additional diagnostic band. ARMS has been successfully used for authentication of several TCM taxa, including *Curcuma* (Sasaki et al., 2002), *Alisma* (Li et al., 2007) and *Rheum* (Yang et al., 2004). The authors emphasize the efficiency and reproducibility of this method. We realize that the ARMS application has its disadvantages as well, i.e., if it is applied to “Goji” products that are processed as powders, one would need to extend the one-step protocol and use both diagnostic primers to examine whether only *Lycium barbarum*, only *Lycium chinense* or both species are present in the respective sample. For strongly degraded DNA that might derive from extraction of processed powder products the amplification of the relatively long psbA-trnH spacer region might be difficult, meaning the internal control that is the advantage of this ARMS approach might suffer in quality. However, most of the commercial “Goji” products are sold as unprocessed dried fruits, for which the presented method easily can be applied. The ARMS approach shown in this study is not limited to superfoods like “Goji,” but equally applicable to other fields of nutrition, and has the potential to be an important tool for consumer safety and quality control when it comes to discriminating “real” ingredients from surrogate species. The public availability of sequences for a large number of different plant taxa in NCBI GenBank makes it easy to design ARMS primers for prospective projects, and in many cases removes the need for initial sequencing to design new ARMS primers. However, we want to emphasize that only sequences from correctly identified herbarium vouchered material should be used for the highly sensitive ARMS method.

## Author Contributions

PN and TH contributed to the idea, topic, background information, and experiment planning. SW carried out the shown experiments under the lab supervision of TH. PN and SW wrote the manuscript.

## Conflict of Interest Statement

The authors declare that the research was conducted in the absence of any commercial or financial relationships that could be construed as a potential conflict of interest.

## Footnotes

1 https://digitalworldbiology.com/FinchTV

## References

[B1] Amagase H. Farnsworth N. R. (2011). A review of botanical characteristics, phytochemistry, relevance in efficacy and safety of *Lycium barbarum* fruit (Goji). *Food Res. Int.* 44 1702–1717. 10.1016/j.foodres.2011.03.027

[B2] Baumann B. Baumann H. Baumann-Schleihauf S. (2001). *Die Kräuterbuch Handschrift des Leonhart Fuchs*, 1st Edn. Stuttgart: Ulmer Verlag.

[B3] Blaschek W. Hänsel R. Keller K. Reichling J. Rimpler H. Schneider G. (1993). *Hagers Handbuch der pharmazeutischen Praxis: Drogen E–O*, Vol. 5 Berlin: Springer Verlag.

[B4] Burke D. S. Smidt C. R. Vuong L. T. (2005). Momordica cochichinensis, *Rosa roxburghii*, wolfberry, and sea buckthorn – highly nutritional fruits supported by tradition and science. *Curr. Top. Nutraceutical. Res.* 3 259–266.

[B5] Chang R. C. So K. F. (2008). Use of anti-aging herbal medicine, *Lycium barbarum*, against aging-associated diseases. What do we know so far? *Cell. Mol. Neurobiol.* 28 643–652. 10.1007/s10571-007-9181-x 17710531 PMC11514989

[B6] Chinadaily (2018). Available at: http://www.chinadaily.com.cn/business/2015-10/20/content_22228738.htm

[B7] Edgar R. C. (2004). MUSCLE: multiple sequence alignment with high accuracy and high throughput. *Nucleic Acids Res.* 32 1792–1797. 10.1093/nar/gkh340 15034147 PMC390337

[B8] Efloras (2018). Available at: http://www.efloras.org/florataxon.aspx?flora_id=2&taxon_id=119146

[B9] Europa (2018). Available at: http://ec.europa.eu/food/safety/novel_food/catalogue/search/public/index.cfm

[B10] EzineArticles (2018). Available at: http://EzineArticles.com/593234

[B11] Felsenstein J. (1985). Confidence limits on phylogenies: an approach using the bootstrap. *Evolution* 39 783–791. 10.1111/j.1558-5646.1985.tb00420.x 28561359

[B12] Fukuda T. (2001). Phylogeny and biogeography of the Genus *Lycium* (Solanaceae): inferences from chloroplast DNA sequences’. *Mol. Phylogenet. Evol.* 19 246–258. 10.1006/mpev.2001.0921 11341807

[B13] Goodwin Z. A. Harris D. J. Filer D. Wood J. R. Scotland R. W. (2015). Widespread mistaken identity in tropical plant collections. *Curr. Biol.* 25 R1057–R1069. 10.1016/j.cub.2015.10.002 26583892

[B14] Hebert P. D. Cywinska A. Ball S. L. deWaard J. R. (2003). Biological identifications through DNA barcodes. *Proc. Biol. Sci.* 270 313–321. 10.1098/rspb.2002.2218 12614582 PMC1691236

[B15] Hollingsworth P. M. Forrest L. L. Spouge J. L. Hajibabaei M. Ratnasingham S. van der Bank M. (2009). A DNA barcode for land plants. *Proc. Natl. Acad. Sci. U.S.A.* 106 12794–12797. 10.1073/pnas.0905845106 19666622 PMC2722355

[B16] Horn T. Barth A. Rühle M. Häser A. Jürges G. Nick P. (2012). Molecular diagnostics of lemon myrtle (*Backhousia citriodora* versus *Leptospermum citratum*). *Eur. Food Res. Technol.* 234 853–861. 10.1007/s00217-012-1688-9

[B17] Horn T. Häser A. (2016). Bamboo tea: reduction of taxonomic complexity and application of DNA diagnostics based on rbcL and matK sequence data. *PeerJ* 4:e2781. 10.7717/peerj.2781 27957401 PMC5149056

[B18] Horn T. Völker J. Rühle M. Häser A. Jürges G. Nick P. (2014). Genetic authentication by RFLP versus ARMS? The case of moldavian dragonhead (*Dracocephalum moldavica* L.). *Eur. Food Res. Technol.* 238 93–104. 10.1007/s00217-013-2089-4

[B19] Konarska A. (2018). Microstructural and histochemical characteristics of *Lycium barbarum* L. fruits used in folk herbal medicine and as functional food. *Protoplasma* 255 1839–1854. 10.1007/s00709-018-1277-2 29948368 PMC6208826

[B20] Kress J. W. Wurdack K. J. Zimmer E. A. Weigt L. A. Janzen D. H. (2005). Use of DNA barcodes to identify flowering plants. *Proc. Natl. Acad. Sci. U.S.A.* 102 8369–8374. 10.1073/pnas.0503123102 15928076 PMC1142120

[B21] Krishnan A. Reshma J. Cyriac A. Sible G. V. (2017). Estimation of genetic diversity in nutmeg (Myristica fragrans Houtt.) selections using RAPD markers. *Int. J. Plant Sci.* 12 102–107. 10.15740/HAS/IJPS/12.2/102-107

[B22] Kumar S. Stecher G. Tamura K. (2016). MEGA7: molecular evolutionary genetics analysis version 7.0 for bigger datasets. *Mol. Biol. Evol.* 33 1870–1874. 10.1093/molbev/msw054 27004904 PMC8210823

[B23] Latimes (2018). Available at: http://articles.latimes.com/2009/aug/05/food/fo-goji5

[B24] Ledford H. (2018). Botanical renaissance. *Nature* 553 396–398. 10.1038/d41586-018-01075-5 29368731

[B25] Levin R. A. Miller J. S. (2005). Relationships within tribe *Lycieae* (Solanaceae): paraphyly of *Lycium* and multiple origins of gender dimorphism’. *Am. J. Bot.* 92 2044–2053. 10.3732/ajb.92.12.2044 21646122

[B26] Levin R. A. Miller J. S. (2009). The utility of nuclear conserved ortholog set II (COSII) genomic regions for species-level phylogenetic inference in *Lycium* (Solanaceae). *Mol. Phylogenet. Evol.* 53 881–890. 10.1016/j.ympev.2009.08.016 19698795

[B27] Li X. Ding X. Chu B. Ding G. Gu S. Qian L. (2007). Molecular authentication of Alisma orientale by PCR-RFLP and ARMS. *Planta Med.* 73 67–70. 10.1055/s-2006-951746 17109255

[B28] Masci A. Carradori S. Casadei M. A. Paolicelli P. Petralito S. Ragno R. (2018). *Lycium barbarum* polysaccharides: extraction, purification, structural characterisation and evidence about hypoglycaemic and hypolipidaemic effects. A review. *Food Chem.* 254 377–389. 10.1016/j.foodchem.2018.01.176 29548467

[B29] Mei Z. Khan M. D. A. Zhang X. Fu J. (2017). Rapid and accurate genetic authentication of *Penthorum* chinense by improved RAPD-derived species-specific SCAR markers. *Biodiversitas* 18 1243–1249. 10.13057/biodiv/d180349

[B30] Mintel (2018). Available at: http://de.mintel.com/pressestelle/deutschland-der-weltweit-zweit-innovativste-markt-fuer-superfoods

[B31] Old J. M. (1992). Detection of mutations by the amplification refractory mutation system (ARMS). *Methods Mol. Biol.* 9 77–84.

[B32] Pang X. Liu C. Shi L. Liu R. Liang D. Li H. (2012). Utility of the trnH-psbA intergenic spacer region and its combinations as plant DNA barcodes: a meta-analysis. *PLoS One* 7:e48833. 10.1371/journal.pone.0048833 23155412 PMC3498263

[B33] Potterat O. (2010). ‘Goji (*Lycium barbarum* and L. chinense): phytochemistry, pharmacology and safety in the perspective of traditional uses and recent popularity’. *Planta Med.* 76 7–19. 10.1055/s-0029-1186218 19844860

[B34] Reinhardt D. Pesce E. R. Stieger P. Mandel T. Baltensperger K. Bennett M. (2003). Regulation of phyllotaxis by polar auxin transport. *Nature* 426 255–260. 10.1038/nature02081 14628043

[B35] Saitou N. Nei M. (1987). The neighbor-joining method: a new method for reconstructing phylogenetic trees. *Mol. Biol. Evol.* 4 406–425. 3447015 10.1093/oxfordjournals.molbev.a040454

[B36] Šamec D. Urliæ B. Salopek-Sondi B. (2018). Kale (Brassica oleracea var. acephala) as a superfood: review of the scientific evidence behind the statement. *Crit. Rev. Food Sci. Nutr.* 10.1080/10408398.2018.1454400 [Epub ahead of print]. 29557674

[B37] Sang T. Crawford D. Stuessy T. (1997). Chloroplast DNA phylogeny, reticulate evolution, and biogeography of *Paeonia* (Paeoniaceae). *Am. J. Bot.* 84 1120–1136. 10.2307/2446155 21708667

[B38] Sasaki Y. Fushimi H. Cao H. Cai S. Q. Komatsu K. (2002). Sequence analysis of Chinese and Japanese curcuma drugs on the 18S rRNA gene and trnK gene and the application of amplification-refractory mutation system analysis for their authentication. *Biol. Pharm. Bull.* 25 1593–1599. 10.1248/bpb.25.1593 12499646

[B39] Schmeil O. Fitschen J. (2011). *Flora Von Deutschland und Angrenzender Länder*, 95th Edn. Wiebelsheim: Quelle & Meyer Verlag.

[B40] Statista (2018a). Available at: https://de.statista.com/infografik/10823/umsatz-mit-superfoods-im-deutschen-lebensmitteleinzelhandel/

[B41] Statista (2018b). Available at: https://de.statista.com/statistik/daten/studie/722141/umfrage/umfrage-zum-regelmaessigen-kauf-von-superfood-in-deutschland/

[B42] SWR (2018). Available at: https://www.swr.de/wissen/gefaelschtes-superfood/-/id=253126/did=21391134/nid=253126/px1w90/index.html

[B43] Sze S. C. Song J. X. Wong R. N. Feng Y. B. Ng T. B. Tong Y. (2008). Application of SCAR (Sequence characterized amplified region) analysis to autheticate *Lycium barbarum* (wolfberry) and its alduterants. *Biotechnol. Appl. Biochem.* 51(Pt 1), 15–21. 10.1042/BA20070096 18052933

[B44] Tanabata T. Shibaya T. Hori K. Ebana K. Yano M. (2012). SmartGrain: high-throughput phenotyping software for measuring seed shape through image analysis. *Plant Physiol.* 160 1871–1880. 10.1104/pp.112.205120 23054566 PMC3510117

[B45] Tate J. A. Simpson B. B. (2003). Paraphyly of tarasa (Malvaceae) and diverse origins of the polyploid species. *Syst. Bot.* 28 723–737.

[B46] Torelli A. Marieschi M. Bruni R. (2014). Authentication of saffron (*Crocus sativus* L.) in different processed, retail products by means of SCAR markers. *Food Control* 36 126–131. 10.1016/j.foodcont.2013.08.001

[B47] Tropicos (2018). Available at: http://www.tropicos.org/Name/29600036?projectid=9

[B48] Untergasser A. Cutcutache I. Koressaar T. Ye J. Faircloth B. C. Remm M. (2012). Primer3 – new capabilities and interfaces. *Nucleic Acids Res.* 40:e115. 10.1093/nar/gks596 22730293 PMC3424584

[B49] Wang J. Ha W. Y. Ngan F. N. But P. P. Shaw P. C. (2001). Application of sequence characterized amplified region (SCAR) analysis to authenticate panax species and their adulterants. *Planta Med.* 67 781–783. 10.1055/s-2001-18340 11731932

[B50] Yang D. Y. Fushimi H. Cai S. Q. Komatsu K. (2004). Polymerase chain reaction restriction fragment length polymorphism (PCR-RFLP) and amplification refractory mutation system (ARMS) analyses of medicinally used Rheum species and their application for identification of Rhei Rhizoma. *Biol. Pharm. Bull.* 27 661–669. 10.1248/bpb.27.661 15133241

[B51] Zhang K. Y. Leung H. W. Yeung H. W. Wong R. N. (2001). Differentiation of *Lycium barbarum* from its related *Lycium* species using random amplified polymorphic DNA’. *Planta Med.* 67 379–381. 10.1055/s-2001-14310 11458465

[B52] Zhonghua Renmin Gongheguo wei sheng bu yao dian wei yuan hui (2000). *Pharmacopoeia of The People’s Republic of China*. Beijing: Chemical Industry Press.

